# The effect of light intensity on microalgae biofilm structures and physiology under continuous illumination

**DOI:** 10.1038/s41598-023-50432-6

**Published:** 2024-01-11

**Authors:** Yan Gao, Olivier Bernard, Andrea Fanesi, Patrick Perré, Filipa Lopes

**Affiliations:** 1 CentraleSupélec, LGPM, Université Paris-Saclay, 91190 Gif-sur-Yvette, France; 2 Inria Sophia Antipolis Méditerranée, Biocore, Université Nice Côte d’Azur, 06902 Valbonne, France; 3 CentraleSupélec, LGPM, CEBB, Université Paris-Saclay, 51110 Pomacle, France

**Keywords:** Biotechnology, Microbiology, Applied microbiology, Biofilms

## Abstract

The interest by biofilm-based microalgae technologies has increased lately due to productivity improvement, energy consumption reduction and easy harvesting. However, the effect of light, one key factor for system’s operation, received less attention than for planktonic cultures. This work assessed the impact of Photon Flux Density (PFD) on *Chlorella vulgaris* biofilm dynamics (structure, physiology, activity). Microalgae biofilms were cultivated in a flow-cell system with PFD from 100 to 500 ${\upmu {\textrm{mol}} \, \textrm{m}^{-2} \, \textrm{s}^{-1}}$. In the first stage of biofilm development, uniform cell distribution was observed on the substratum exposed to 100 ${\upmu \textrm{mol} \, \textrm{m}^{-2} \, \textrm{s}^{-1}}$ while cell clusters were formed under 500 ${\upmu \textrm{mol} \, \textrm{m}^{-2} \, \textrm{s}^{-1}}$. Though similar specific growth rate in exponential phase (ca. 0.3 ${\textrm{d}^{-1}}$) was obtained under all light intensities, biofilm cells at 500 ${\upmu \textrm{mol} \, \textrm{m}^{-2} \, \textrm{s}^{-1}}$ seem to be ultimately photoinhibited (lower final cell density). Data confirm that *Chlorella vulgaris* showed a remarkable capability to cope with high light. This was marked for sessile cells at 300 ${\upmu \textrm{mol} \, \textrm{m}^{-2} \, \textrm{s}^{-1}}$, which reduce very rapidly (in 2 days) their chlorophyll-a content, most probably to reduce photodamage, while maintaining a high final cell density. Besides cellular physiological adjustments, our data demonstrate that cellular spatial organization is light-dependent.

## Introduction

Microalgae are nowadays considered a promising resource for food, feed and high-value biocompounds production, and in the long term, for biofuels generation^1^. Currently, microalgae are mainly cultivated in suspension using either open systems (raceways) or closed reactors (photobioreactors)^2,3^. Raceways show advantages for microalgae cultivation compared to closed systems since they require less energy, their construction is cheaper and operational costs are lower. They are the most widespread at industrial scale^3^. The productivity of these suspension-based systems stays however low due to reduced biomass resulting from inefficient light penetration, low ${\hbox {CO}_{2}}$ transfer rate, non-efficient mixing and non-sterile conditions. They also use large amounts of water and require large land area^3,4^. Besides, in suspended cultures, biomass is diluted (less than 1%) so that harvesting is energy and cost demanding^4^.

A considerable interest by biofilm-based systems has been reported lately^5^ due to their higher footprint productivity^4,6^ and reduced costs of harvesting and dewatering^7,8^. Many photobioreactors with different configurations have been proposed and are described in Wang et al.^5^. By contrast to conventional systems, a higher effective light penetration and ${\hbox {CO}_{2}}$ assimilation rate explain productivity improvements of biofilm-based systems^4,9,10^. For instance, 100% effective illumination was reported in *S. dimorphus* biofilms (biomass density of 107.6 ${\textrm{g}\, \textrm{m}^{-2}}$), while only 31.1% was recorded in a conventional open-pond (biomass density of 90 ${\textrm{g}\, \textrm{m}^{-2}}$), both exposed to the same light conditions^11^. Besides, a more efficient ${\hbox {CO}_{2}}$ transfer in biofilm than in suspension has been reported by Huang et al.^6^ with biomass areal density improvement.

Though the increasing interest by algal biofilm-based systems, they are still an emergent and immature technology for which much remains to be understood^12^. Unlike planktonic cells that are suspended in the medium and subjected to mixing, microalgae biofilms are regarded as a slimy layer of microalgae that attach and grow on solid surfaces, presenting a 3D structure (spatial arrangement of the cells, polymers and voids) and with features strongly differing from their planktonic counterparts^13^. Biofilms are highly heterogeneous in time and space. They are characterized by high cell density, an extracellular polymer substances (EPS) matrix with physical, chemical, biological and metabolic heterogeneities^13^ over depth. 3D structure has been shown to be strongly affected by environmental and operation factors such as shear stress^14^, nutrients transport^15–17^, and it is also species-dependent^18^. However, the impact of light (quality, quantity) on photosynthetic biofilm growth, structure, cell physiology and regulation is largely unknown.

Light is a critical factor for microalgae growth. For planktonic cultures, the optimal Photon Flux Density (PFD) ranges in general from 100 to 400 ${\upmu \textrm{mol} \, \textrm{m}^{-2} \, \textrm{s}^{-1}}$, depending on the species^5,19–21^. Higher light intensities damage the photosynthetic apparatus. Interestingly, algae have the ability to photoacclimate to different light conditions in order to maximize the photosynthetic efficiency and reduce photodamage. Physiological changes are then triggered by the cell, such as the modification of photosystem size or chlorophyll content^22^. It is widely known that chlorophyll-a content decreases with intensified irradiance as a strategy of self-protection to cope with light stress. The maximum quantum yield of PSII ($F_v/F_m$) derived from variable fluorescence measurements is an index of the health status of microalgal cells, indicating if there is light or nutrient stress on PSII^23–25^. Healthy microalgal cultures have $F_v/F_m$ values in the range of 0.7–0.8^26^, whereas a reduction in $F_v/F_m$ suggests a decrease in PSII photochemistry efficiency or a disorder in or damage to the photosynthetic apparatus^27^. Algal cells can also adjust cellular composition in response to light changes, like carbon and lipid contents^28,29^. Other properties such as cell volume are also affected by light, being positively correlated to PFD^28,30,31^.

However, unlike planktonic cultures, the impact of light intensity on biofilm growth, structure, cell physiology and regulation is poorly studied. In this work, we therefore assessed biofilm dynamics under four light intensities, ranging from 100 to 500 ${\upmu \textrm{mol} \, \textrm{m}^{-2} \, \textrm{s}^{-1}}$. 3D structure of biofilms, cell physiological adjustments (such as photoacclimation) and metabolic activity (photosynthesis and dark respiration) were measured. This is of paramount importance to better understand the overall functioning of photosynthetic biofilms in order to further exploit them efficiently in bioproduction.

## Results and discussion

### Physiological shift of microalgae cells from planktonic to sessile state

**Figure 1:**
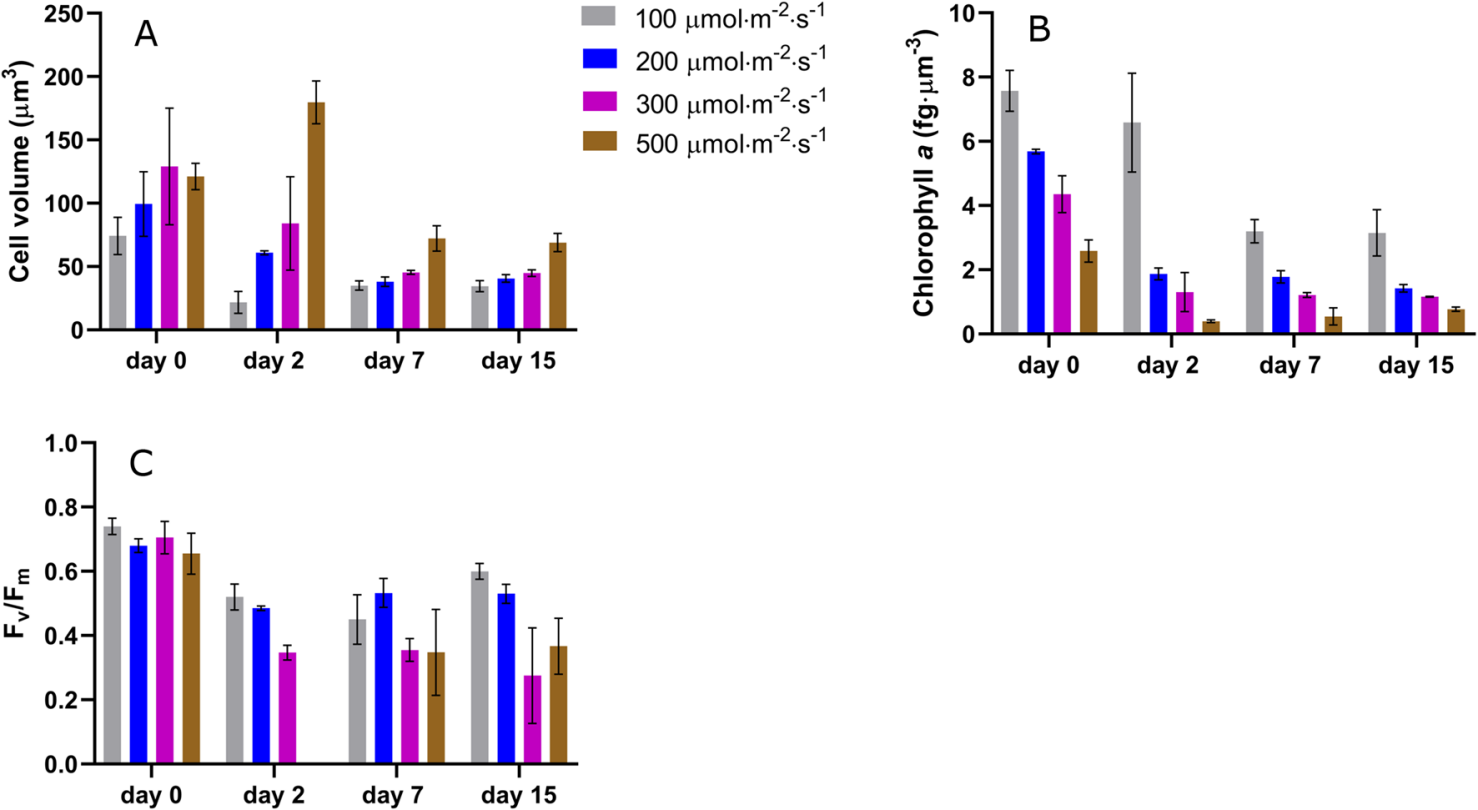
Evolution of cell physiological parameters over time in biofilms developed at different light conditions. Day 0 refers to the inoculum culture (**A**) cell volume; (**B**) chlorophyll-a content; (**C**) $F_v/F_m$.

A sharp decrease in cell volume was observed for all light conditions during the first 2 days after inoculation, except for 500 ${\upmu \textrm{mol} \, \textrm{m}^{-2} \, \textrm{s}^{-1}}$ which presents a significant increase (Fig. 1A). The same trend was detected for the chlorophyll content and the maximum quantum yield (Fig. 1B,C). This suggests a physiological acclimation of the cells when switching from planktonic to sessile state, potentially triggered by changes in the environmental conditions, as proposed by Li et al.^32^. Similarly, Wang et al.^11^ observed a reduction in the chlorophyll content (60%) of *Scenedesmus dimorphus* biofilms in only 2 days after inoculation. Lan et al.^33^ also suggested that changes in environmental conditions could be likely responsible for a shift in $F_v/F_m$ values of *Microcoleus vaginatus* cells moving from planktonic to sessile state. Indeed, even if the average PFD supplied to the two systems was similar, differences in light quantity and quality may have occurred in suspension and biofilm cultures (see Supplementary Fig.S1 online). While planktonic cells (inoculum, $t_0$) undergo fluctuating light due to auto-shading and agitation, biofilm cells are submitted to constant PFD. It should be pointed out that other factors already reported for bacterial biofilms, such as mechanical stress and/or quorum sensing^34,35^, could also be at play.

### Biofilm dynamics: biomass and physiological properties

**Figure 2:**
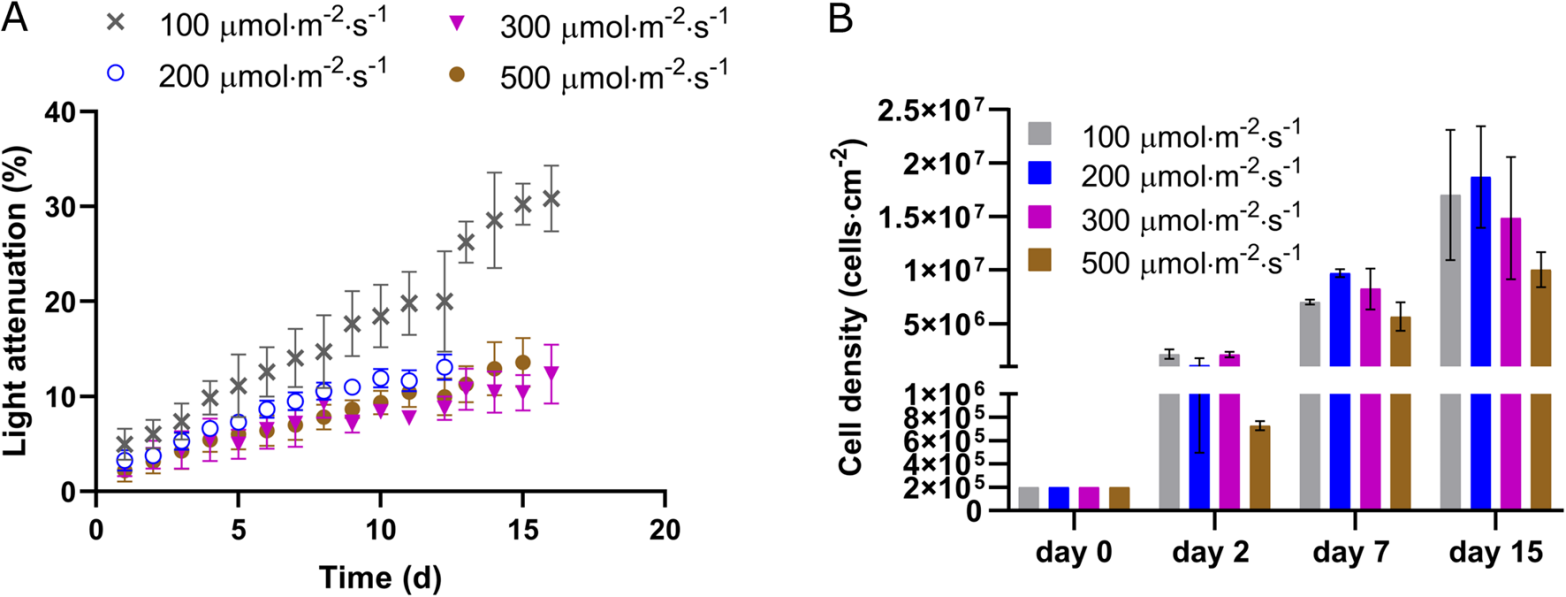
(**A**) Light attenuation dynamics; (**B**) Areal cell density dynamics under 100, 200, 300, 500 ${\upmu \textrm{mol} \, \textrm{m}^{-2} \, \textrm{s}^{-1}}$.

PFD from 100 to 300 ${\upmu \textrm{mol} \, \textrm{m}^{-2} \, \textrm{s}^{-1}}$ were optimal for *Chlorella vulgaris* biofilm growth (Fig. 2A,B). This range is in agreement with other studies described in the literature, reporting saturating rates in the range of 200–280 ${\upmu \textrm{mol} \, \textrm{m}^{-2} \, \textrm{s}^{-1}}$ for *Chlorella* sp. biofilms^6,36,37^. The dynamics of light attenuation and cell density for all the light conditions investigated in this study are shown in Fig. 2. Both parameters show a biomass increase due to biofilm growth under all light conditions. The highest light attenuation (30%) was obtained at day 15 in biofilms under 100 ${\upmu \textrm{mol} \, \textrm{m}^{-2} \, \textrm{s}^{-1}}$ while those exposed to 200, 300 and 500 ${\upmu \textrm{mol} \, \textrm{m}^{-2} \, \textrm{s}^{-1}}$ only attenuated 10% of the light. Interestingly, this marked difference in light attenuation was not due to a significant difference in cell density. Indeed, similar cell densities were observed at day 15 for biofilms exposed to light in the range of 100–300 ${\upmu \textrm{mol} \, \textrm{m}^{-2} \, \textrm{s}^{-1}}$ ($p > 0.05$). Besides, a significantly lower value is measured for 500 ${\upmu \textrm{mol} \, \textrm{m}^{-2} \, \textrm{s}^{-1}}$ ($p<$ 0.05) compared to that at 200 ${\upmu \textrm{mol} \, \textrm{m}^{-2} \, \textrm{s}^{-1}}$ while similar light attenuation is reported. These results suggest that light attenuation is not only dependent on cell density but also on chlorophyll content and cell size. Under low light conditions (at 100 ${\upmu \textrm{mol} \, \textrm{m}^{-2} \, \textrm{s}^{-1}}$), microalgae tend to over-accumulate chlorophyll-a that in packed layers of cells could lead to a stronger self-shading and light attenuation, compared to high light conditions (Fig. 1B). On the other hand, the lower chlorophyll content observed for the strongest PFD of 300 and 500 ${\upmu \textrm{mol} \, \textrm{m}^{-2} \, \textrm{s}^{-1}}$, likely in response to mitigate the light stress, made the biofilms more optically transparent leading to a higher transmittance of light.

The dynamics of sessile cell volume and chlorophyll content (Fig. 1) are characterized by a general decreasing trend from day 2 to day 7. Afterwards, these values leveled-off, revealing full photoacclimation. A fast decrease in chlorophyll content, over only 2 days, occurred for light intensities higher than 100 ${\upmu \textrm{mol} \, \textrm{m}^{-2} \, \textrm{s}^{-1}}$ while 7 days were required for 100 ${\upmu \textrm{mol} \, \textrm{m}^{-2} \, \textrm{s}^{-1}}$. The time required to achieve photoacclimation seems thus to be light dependent. Similar results are reported in the literature. Photoacclimation was also observed in *Scenedesmus dimorphus* and *Chlorella vulgaris* biofilms over 10 days cultivation period^11^. In another work, *Chlorella vulgaris* biofilms presented a decrease in the chlorophyll content in only 2 days when exposed to PFD conditions ranging from 40 to 280 ${\upmu \textrm{mol} \, \textrm{m}^{-2} \, \textrm{s}^{-1}}$^6^.

After a first decline, the maximum quantum yield of PSII, ($F_v/F_m$ ratio), increased slightly from day 7 to day 15 for biofilms under 100 ${\upmu \textrm{mol} \, \textrm{m}^{-2} \, \textrm{s}^{-1}}$. Our results are in agreement with the work of Lan et al.^33^ who observed a decrease of $F_v/F_m$ of *Microcoleus vaginatus* cells to (0.1–0.2) after inoculation, then recovering to 0.6 in 10–15 days^33^.

It also appears that biofilms exposed to 300 and 500 ${\upmu \textrm{mol} \, \textrm{m}^{-2} \, \textrm{s}^{-1}}$ are the most stressed ($F_v/F_m$ < 0.4) (Fig. 1C). This is explained by the combined effects of light intensity and low biofilm development (only approximately 10% of the incident light was attenuated for the higher light intensities). Our observations are consistent with data from Wang et al.^37^ who investigated the effect of light on the photosynthetic activity of a *Chlorella* sp. biofilm exposed to irradiances ranging from 20 to 400 ${\upmu \textrm{mol} \, \textrm{m}^{-2} \, \textrm{s}^{-1}}$. Wang et al. found $F_v/F_m$ value higher than 0.65 with PFD of 100 ${\upmu \textrm{mol} \, \textrm{m}^{-2} \, \textrm{s}^{-1}}$, but a significant decline was obtained when PFD reached 200 ${\upmu \textrm{mol} \, \textrm{m}^{-2} \, \textrm{s}^{-1}}$ and 400 ${\upmu \textrm{mol} \, \textrm{m}^{-2} \, \textrm{s}^{-1}}$ ($F_v/F_m$ <0.6), suggesting light stress^37^. Li et al.^36^, measured a $F_v/F_m$ value of 0.56 for biofilms of *Chlorella vulgaris* exposed to 500 ${\upmu \textrm{mol} \, \textrm{m}^{-2} \, \textrm{s}^{-1}}$. This higher value may be explained by differences in cell density and/or cultivation conditions (flow and light quality). Indeed, the inoculum cell density was much higher than that of our study (20–100 times). This ensures an increased attenuation of light, protecting cells from photo-inhibition.

**Figure 3:**
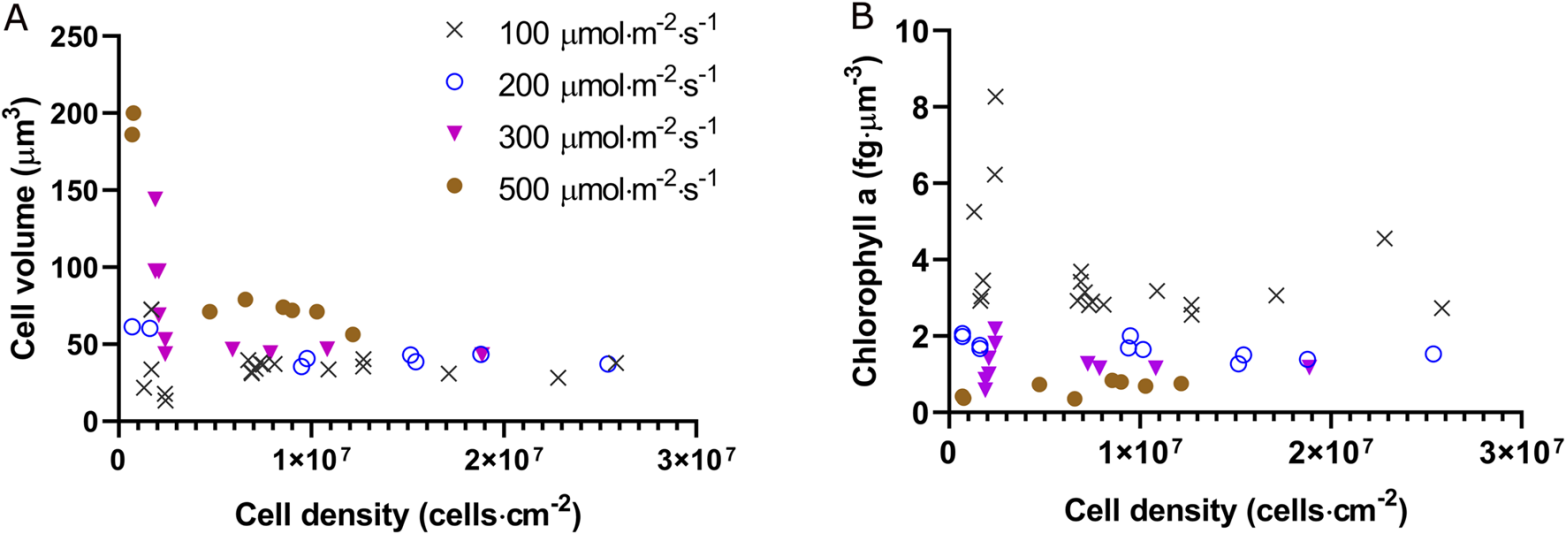
Cell volume (**A**), and chlorophyll-a content (**B**) over cell density.

### Effect of light intensity on biofilm growth, metabolism and physiology

A mean growth rate of 0.3 ${\textrm{d}^{-1}}$ was measured for all the PFDs tested (Table 1). Regardless of the light intensity, exponential growth was observed during 4–6 days, and was linear afterwards. Higher growth rates were though determined for *Chlorella* sp. biofilms in the literature: 0.45 ${\textrm{d}^{-1}}$ and 0.8 ${\textrm{d}^{-1}}$ in the work of Fanesi et al.^14,18^, 0.4–0.5 ${\textrm{d}^{-1}}$ in the study of Yuan et al.,^38^. In another work^39^, *Chlorella vulgaris* biofilm growth rate even reached 1.2 ${\textrm{d}^{-1}}$. This divergence can be explained by several differences in the experimental set-up including growth substratum, hydrodynamics and inoculum size. Growth substratum properties (roughness, hydrophobicity, etc.,) are known to play an important role in cell adhesion^40^. Unlike other studies where porous filtration membranes or other rough materials were used^38,39^, a smooth glass surface was used here to observe in situ biofilm development. Also, hydrodynamics strongly affect biofilm development^18^. Different from other works^18,38,39^, a constant shear stress of 2.3 ${\textrm{mPa}}$ was applied in our study, which may have contributed to a continuous cell detachment, decreasing the net growth rate of biofilms^14,41^. Finally, it has also been reported that the inoculum size influences biofilm growth and development^6,36^.

**Table 1:** Maximum specific growth rate of biofilms exposed to different light conditions.

PFD (${\upmu \textrm{mol} \, \textrm{m}^{-2} \, \textrm{s}^{-1}}$)	100	200	300	500
$\mu _l$ (${\textrm{d}^{-1}}$)	0.32 ± 0.10	0.30 ± 0.11	0.29 ± 0.05	0.28 ± 0.08

The growth rate is expressed as the mean value ± standard deviation.

**Figure 4:**
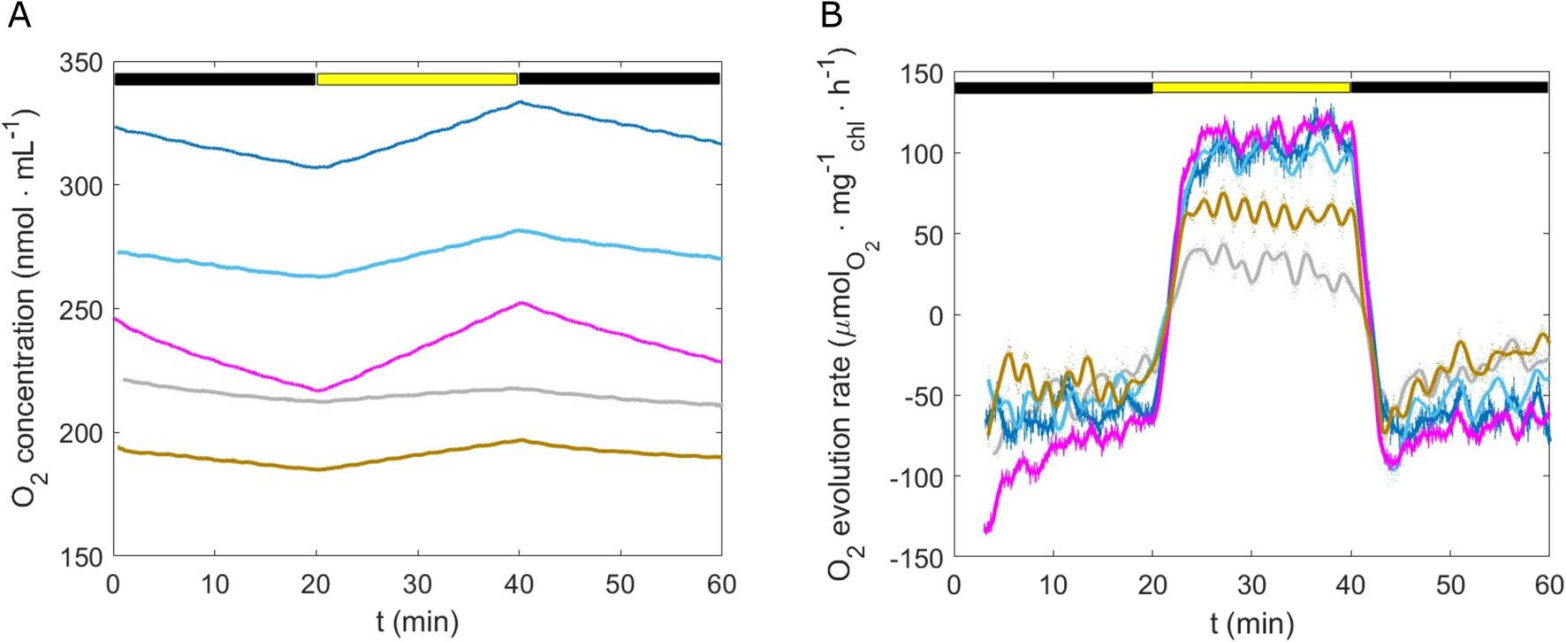
(**A**) ${\hbox {O}_{2}}$ concentration over time under light (yellow bar) and dark (black bar, 20 ${\textrm{min}}$) phases for biofilms at day 7 developed at different light regimes. (**B**) ${\hbox {O}_{2}}$ evolution rate per chlorophyll-a over time, extracted from (**A**). 100 ${\upmu \textrm{mol} \, \textrm{m}^{-2} \, \textrm{s}^{-1}}$: grey line; 200 ${\upmu \textrm{mol} \, \textrm{m}^{-2} \, \textrm{s}^{-1}}$: two blue curves represent two replicates; 300 ${\upmu \textrm{mol} \, \textrm{m}^{-2} \, \textrm{s}^{-1}}$: magenta line; 500 ${\upmu \textrm{mol} \, \textrm{m}^{-2} \, \textrm{s}^{-1}}$: brown line.

The constant behavior of the growth rate with respect to light intensity could be explained by the physiological responses enacted by the cells. Different strategies are used by the cells to cope with excess of light and improve or maintain growth, including a chlorophyll content decrease and/or build-up of storage pools (e.g. carbohydrates, lipids) and changes in photosynthetic activity. In line with the state of the art on photoacclimation for suspended cultures^31,42^, the highest chlorophyll-a content was observed when the biofilms were cultivated at the lowest PFD (100 ${\upmu \textrm{mol} \, \textrm{m}^{-2} \, \textrm{s}^{-1}}$) (Fig. 3, $p<0.05$, Figure S2). For light intensities of 200 and 300 ${\upmu \textrm{mol} \, \textrm{m}^{-2} \, \textrm{s}^{-1}}$, the content in chlorophyll-a was half of that observed at 100 ${\upmu \textrm{mol} \, \textrm{m}^{-2} \, \textrm{s}^{-1}}$. A similar behavior has been reported by Huang et al.,^6^ who observed a decreasing chlorophyll content when microalgae biofilms where subjected to increasing PFD. We have to point out that since cells were bigger at higher light intensities, chlorophyll was more diluted in the cells. Also, when considering similar cell densities, it appears that photoacclimated microalgae presented larger cell volume at 500 ${\upmu \textrm{mol} \, \textrm{m}^{-2} \, \textrm{s}^{-1}}$ compared to those cultivated at 100 ${\upmu \textrm{mol} \, \textrm{m}^{-2} \, \textrm{s}^{-1}}$, ($p<0.05$, Fig. 3, Figure S2). On the other hand, no significant difference was detected among the other light intensities. Only few reports discussed the effect of light intensity on sessile cell size. Zhang et al.^43^ observed that cell diameter remained at 3.5 ${\upmu \textrm{m}}$ in a biofilm of *Chlorella vulgaris*, when grown at low light intensities (50–104 ${\upmu \textrm{mol} \, \textrm{m}^{-2} \, \textrm{s}^{-1}}$). In agreement with our results, the literature on planktonic microalgae describes a positive correlation between cell volume and PFD^30,31,44^. This relationship can be explained by the accumulation of photosynthetic products (especially carbohydrates) at high PFD as a sink of electrons and carbon when cells are subjected to excessive excitation energy^28^. Moreover, larger cells are generally considered to be less subjected to photo-inhibition due to lower effective cross-sections^45,46^. Finally, compared to the biofilms grown at 100 ${\upmu \textrm{mol} \, \textrm{m}^{-2} \, \textrm{s}^{-1}}$, those exposed to 300 ${\upmu \textrm{mol} \, \textrm{m}^{-2} \, \textrm{s}^{-1}}$ presented higher metabolic activity (net photosynthetic and dark respiration rates were fourfold (112 ${\upmu \textrm{mol}_{\textrm{O}_2} \, \textrm{mg}^{-1}_{\textrm{chl}} \, \textrm{h}^{-1}}$) and twofold (72 ${\upmu \textrm{mol}_{\textrm{O}_2} \, \textrm{mg}^{-1}_{\textrm{chl}} \, \textrm{h}^{-1}}$), respectively) (Fig. 4, Figure S3), lower chlorophyll content and maximum quantum yield. These results suggest that the cells at high light were under a high excitation pressure and responded by decreasing the amount of absorbed energy (lower chlorophyll and greater volume) and maximized their carbon fixation capacity (higher photosynthetic capacity).

Nevertheless, at 500 ${\upmu \textrm{mol} \, \textrm{m}^{-2} \, \textrm{s}^{-1}}$, the $F_v/F_m$ and the lower photosynthetic activity suggest that these physiological responses were not enough to compensate for the high excitation pressure and photoinhibition occurred (a decrease of 40% of the net photosynthetic rate was measured, Fig. 4, Figure S3). Further experiments should be carried out to better understand protective mechanisms in photosynthetic biofilms. Parameters such as non-photochemical quenching (NPQ), xanthophylls and intracellular compounds (lipids, carbohydrates) should be measured to better understand the transition towards photoinhibition.

### Biofilm structure

**Figure 5:**
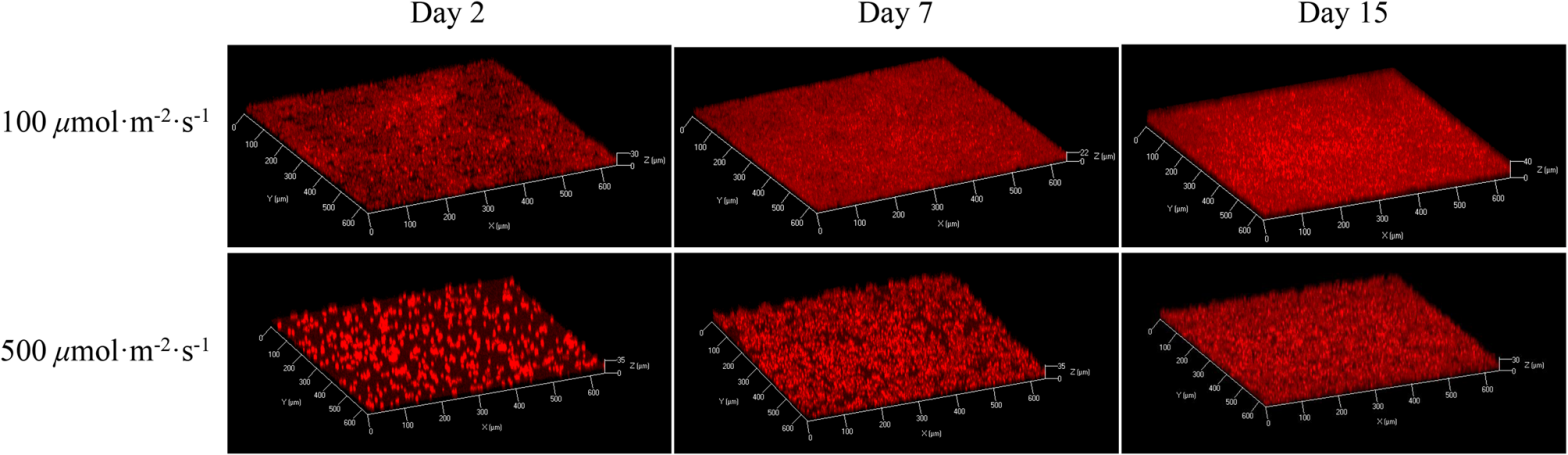
3D structure of biofilms under continuous light 100 and 500 ${\upmu \textrm{mol} \, \textrm{m}^{-2} \, \textrm{s}^{-1}}$ at day 2, 7, and 15.

**Figure 6:**
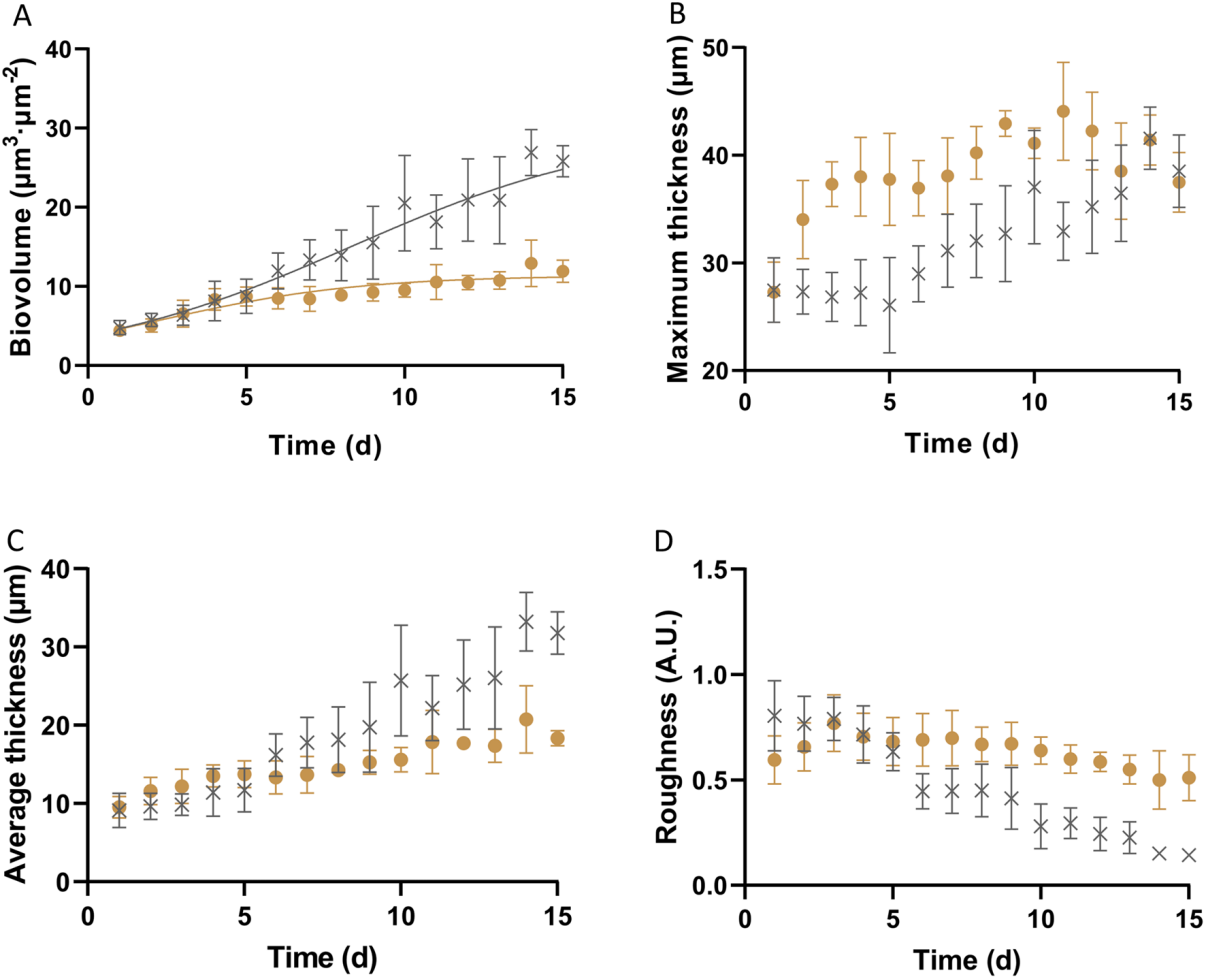
Structural parameters (Biovolume, Maximum thickness, Average thickness, and Roughness) dynamics under continuous light of 100 (gray cross symbols) and 500 ${\upmu \textrm{mol} \, \textrm{m}^{-2} \, \textrm{s}^{-1}}$ (brown solid dots), respectively.

Representative CLSM stacks and the structural parameters obtained from image processing are reported as a function of time in Figs. 5 and 6 for 100 and 500 ${\upmu \textrm{mol} \, \textrm{m}^{-2} \, \textrm{s}^{-1}}$. Biovolume and average thickness increased over time in biofilms exposed to both light intensities (Fig. 6A), in agreement with results of other studies at similar PFD^18^. This is also consistent with cell density dynamics shown in Fig. 2B. Inhibition due to high light was also confirmed by the structural parameters obtained from the CLSM stacks (biovolume 2 fold lower at day 15, $p<0.05$) despite similar specific growth rates (Table 1).

Interestingly, the maximum thickness depicts different patterns under the two PFDs. At 100 ${\upmu \textrm{mol} \, \textrm{m}^{-2} \, \textrm{s}^{-1}}$, the maximum thickness was maintained stable at 27 ${\upmu \textrm{m}}$ during the initial 4 days and then increased gradually until the plateau at around 40 ${\upmu \textrm{m}}$. On the other hand, for 500 ${\upmu \textrm{mol} \, \textrm{m}^{-2} \, \textrm{s}^{-1}}$, the maximum thickness rapidly increased from 27 to 37 ${\upmu \textrm{m}}$ surpassing the values observed at 100 ${\upmu \textrm{mol} \, \textrm{m}^{-2} \, \textrm{s}^{-1}}$ in only 3 days, and then leveled-off. These observations demonstrate that the spatial arrangement of sessile cells is light dependent. Since biofilms initiated from sparse cell density (ca. 15 ${\upmu \textrm{m}}$ distant from each other), the behavior under 100 ${\upmu \textrm{mol} \, \textrm{m}^{-2} \, \textrm{s}^{-1}}$ implies that cells divided and dispersed evenly on the substratum, but no clear growth occurred in the z-dimension (no increase in thickness). Afterwards, the substratum was fully covered and an increase in thickness was observed. An interesting clustering behavior was observed in the biofilms exposed to 500 ${\upmu \textrm{mol} \, \textrm{m}^{-2} \, \textrm{s}^{-1}}$ which formed colonies as confirmed by the CLSM 3D reconstructions (Fig. 5). Colonies formation was thus the reason for the sharp increase in maximum thickness during the early step of biofilm formation (Fig. 6B). Afterwards, voids between cell clusters were filled up through cell division and thickness kept roughly constant until the end of the assay. These results are in agreement with the roughness coefficient plotted in Fig. 6D. Biofilms at 100 ${\upmu \textrm{mol} \, \textrm{m}^{-2} \, \textrm{s}^{-1}}$ showed a decreasing trend, suggesting that they got smoother with time. Instead, in the latter stages of biofilm development, roughness at 500 ${\upmu \textrm{mol} \, \textrm{m}^{-2} \, \textrm{s}^{-1}}$ is higher than at 100 ${\upmu \,\textrm{mol} \, \textrm{m}^{-2} \, \textrm{s}^{-1}}$ (Fig. 6D).

From our findings, it is evident that the way cells spatially organize likely represents another strategy used by biofilm-forming microalgae to promptly react to local conditions^16,43,47^, and especially to PFD. Indeed, we were able to show that physiological and structural responses co-occur when biofilm cells are exposed to high PFD. Cells under 500 ${\upmu \textrm{mol} \, \textrm{m}^{-2} \, \textrm{s}^{-1}}$ react very rapidly (2–3 days) to light by increasing their size (Fig. 1A), decreasing the chlorophyll content (Fig. 1B), dividing and organizing themselves in colonies instead of spreading on the substratum (Fig. 5). They are thus able to divide exponentially as cells exposed to lower light (Table 1). Nevertheless, those physiological and structural adaptations are not enough to completely avoid photoinhition as suggested by the lower photosynthetic rate and the lower cell number produced at the end of the assays under 500 ${\upmu \textrm{mol} \, \textrm{m}^{-2} \, \textrm{s}^{-1}}$. More experiments, combined with modelling approaches, are though required to fully understand this physiological and structural adaptation.

## Material and methods

### Microalgae species and inoculum culture

*Chlorella vulgaris* SAG 211-11B (Göttingen, Germany) was cultivated in 3N-Bristol medium^48^. Inocula cultures were cultivated in a 100 ${\textrm{mL}}$ glass tube with a working volume of 70 ${\textrm{mL}}$ in a PSI MC1000 multicultivator (Photon systems instruments, Drásov, Czech Republic) at 25 ℃ with constant aeration by bubbling. Cultures were pre-acclimated to each light condition for 2 weeks (see subsection “Biofilm system set-up”) and maintained in exponential phase (cell concentration: 2–3 ${\times 10^6 \textrm{cells} \, \textrm{mL}^{-1}}$) by frequent dilutions (every 2–3 days).

### Biofilm system set-up

**Figure 7:**
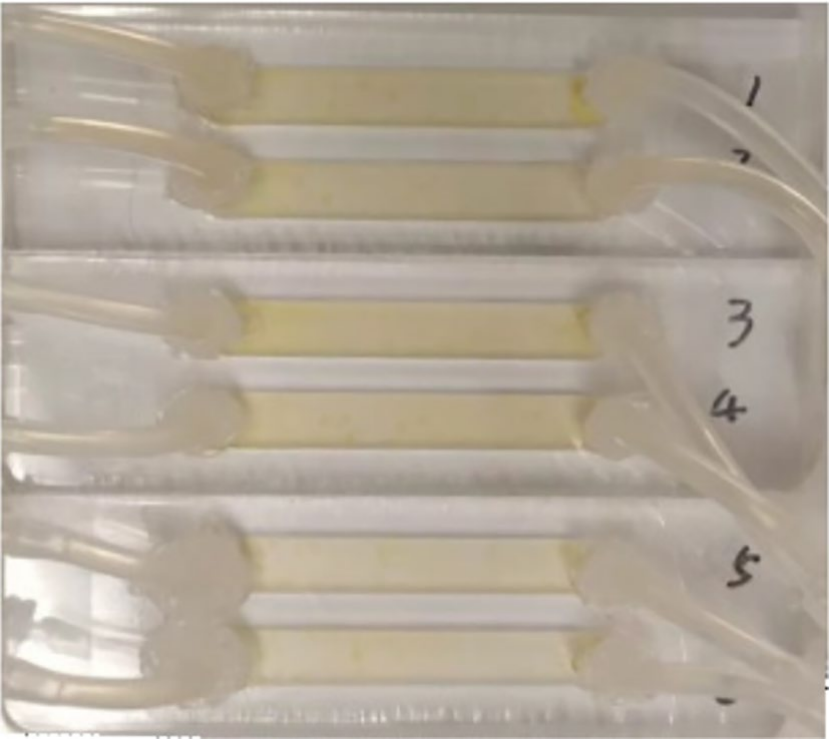
Custom-made flow-cell with *C. vulgaris* biofilms growing inside. Each one has two separated channels.

*C. vulgaris* biofilms were cultivated in a custom-made flow cell of Poly-methyl methacrylate (PMMA) (${40\,\textrm{mm} \times 6\, \textrm{mm} \times 3\, \textrm{mm}}$ in length, width and height, respectively), with a cover glass as substratum (Fig. 7). The set-up has been already described by Le Norcy et al.^49^ and Fanesi at al.^14^. Before inoculation, the system was first sterilized by sodium hypochlorite solution (0.5%, 0.1 ${\textrm{mL}\, \textrm{min}^{-1}}$) for 3 ${\textrm{h}}$ and then flushed with autoclaved distilled water. It was finally filled by 3N-Bristol medium overnight. To avoid bubbles development, the system was equilibrated with a flow rate of 0.1 ${\textrm{mL}\, \textrm{min}^{-1}}$ throughout sterilization, washing and medium filling procedures. For inoculation, 3 ${\textrm{mL}}$ pre-diluted culture (7${\times 10^5 \textrm{cell} \, \textrm{mL}^{-1}}$) was injected into each channel through an in-line luer injection port (Ibidi GmbH, Germany). After 24 h without flow to ensure cell attachment, fresh medium (laminar flow regime) was added to the flow-cell. The shear stress ($\tau$, ${\textrm{mPa}}$) is estimated by equation (1) assuming the channel width to be significantly larger than its height^50^:

1 $$\begin{aligned} \tau = \frac{6Q\mu }{wh^2} \end{aligned}$$

where *Q* is the flow rate (${\upmu \textrm{L}\, \textrm{s}^{-1}}$), $\mu$ is the dynamic viscosity of water (0.91 ${\textrm{mPa} \, \textrm{s}^{-1}}$) at 24 ℃, *w* and *h* are the width (${\textrm{mm}}$) and height (${\textrm{mm}}$) of the flow-cell channel, respectively.

The flow parameters were the following: flow rate of 0.1 ${\textrm{mL} \, \textrm{min}^{-1}}$, velocity of 0.093 ${\textrm{mm}\, \textrm{s}^{-1}}$, Reynolds number of 0.37, and shear stress of 2.3 ${\textrm{mPa}}$. The temperature was controlled at $24\pm 1$ ℃.

Light was continuously supplied to the culture at 106, 200, 310 and 496 ± 3 ${\upmu \textrm{mol} \, \textrm{m}^{-2} \, \textrm{s}^{-1}}$, respectively. They are denoted as 100, 200, 300, 500 ${\upmu \textrm{mol} \, \textrm{m}^{-2} \, \textrm{s}^{-1}}$, respectively. Light was provided by a LED system (Light Emitting Diode, Alpheus LED, Montgeron, France) with parameters controlled by the software Ether controller (v6.6.0.2). The light spectra information is provided in Supplementary data (400–500 ${\textrm{nm}}$: 30%, 500–600 ${\textrm{nm}}$: 21%, 600–700 ${\textrm{nm}}$: 48%, 700–800 ${\textrm{nm}}$: 1%). PFD was measured by a Quantitherm PAR/Temp Sensor (Hansatech Instruments Ltd, Norfolk, The UK). The number of independent assays were 5, 4, 3, 4 for 100, 200, 300 and 500 ${\upmu \textrm{mol} \, \textrm{m}^{-2} \, \textrm{s}^{-1}}$, respectively. For each independent assay, 6 channels were run as replicates.

### Physiological parameters

Physiological parameters (cell volume, chlorophyll-a content, maximum quantum yield of PSII-$F_v/F_m$) were assessed by off-line measurements on day 2, 7, and 15, respectively, by extracting the cells from each channel. Physiology of the inoculum culture (day 0) was also analyzed to compare with that of biofilm cells.

#### Cell volume

Cell volume was measured by microscope imaging (Brightfield in transmission mode) and subsequent image analysis (software ImageJ v1.48). On day 2, due to the low cell density direct observation could be done and the cell volume was measured in *situ*. For longer times, the cells were withdrawn, concentrated (to $1 \times 10^8 - 2 \times 10^8 {\textrm{cells} \, \textrm{mL}^{-1}}$ by centrifugation at 14.5 ${\textrm{krpm}}$) and observed by optical microscopy. 2D images were first obtained by the inverted Zeiss LSM 700 Confocal Laser Scanning Microscope (CLSM, Carl Zeiss microscopy GmbH, Jena, Germany) with Zen 10.0 software black edition (Carl Zeiss microscopy GmbH, Jena, Germany). LD Plan-Neofluar 20 $\times$ 0.4 Korr M27 objective with a 0.4 N.A. was used to take the picture with a frame size of 256 $\times$ 256 pixels (pixel size: 0.32 ${\upmu \textrm{m}}$) and image size of 82.2 $\times$ 82.2 ${\upmu \textrm{m}^2}$. On the other hand, optical track channel (TV1) was used for optical microscopy acquisition. The 2D image was analysed by ImageJ v1.48 software directly. The image type was set to 8-bit before thresholding. After making binary of the image and all cells being filled in black with a white background, the area of each cell was estimated. The cell size limit was set as 0- infinity with the pixel units concerned. Assuming all cells to be spheres of similar diameter, the cell volume can be determined from the cell area (Eq. (2)):

2 $$\begin{aligned} Cell \; volume = \frac{4}{3} \, A \, \sqrt{\frac{A}{\pi }} \end{aligned}$$

where *A* (${\upmu \textrm{m}^2}$) is the area of the microalgae cell in the 2D-image.

#### Chlorophyll-a content

Chlorophyll-a was extracted in DMSO (Dimethyl-sulphoxide) according to (Wellburn 1994)^51^. First, cells (range: $4 \times 10^6$–$10 \times 10^6$ cells) were filtrated on glass fiber filters (Fisher Scientific, size: 47 ${\textrm{mm}}$, EU). The filter was cut into 5 ${\textrm{mm}}$ strip and then submerged in 1 ${\textrm{mL}}$ DMSO. Chlorophyll-a extraction was carried out for 40 ${\textrm{min}}$ at room temperature in the dark. After being centrifuged for 5 ${\textrm{min}}$ with 1300 ${\textrm{rpm}}$, the supernatant was transferred to a 1.5 ${\textrm{mL}}$ cuvette for absorbance measurement by a UV Visible Spectrophotometer (Thermo Fisher Scientific, EVOLUTION 60s, China). Chlorophyll-a (${\upmu \textrm{g} \, \textrm{mL}^{-1}}$) was calculated with Eq. (3):

3 $$\begin{aligned} Chlorophyll-a = 12.19 \cdot abs665-3.45 \cdot abs649 \end{aligned}$$

where abs665 and abs649 refer to the absorption at wavelength 665 ${\textrm{nm}}$ and 649 ${\textrm{nm}}$, respectively. Chlorophyll-a content per cell volume (${\textrm{fg} \, \upmu \textrm{m}^{-3}}$) was then calculated.

#### Maximum quantum yield of PSII

The maximum quantum yield of PSII ($F_v/F_m$) of re-suspended biofilm cells was measured by a portable pulse amplitude modulation (PAM) fluorometer (AquaPen, Photon Systems Instruments, AP110C, Czech Republic, software FluorPen v1.0.1.8). According to the chlorophyll-a content, the cell concentration was adjusted to the range of $5 \times 10^5$–$1 \times 10^6$ ${\textrm{cells} \, \textrm{mL}^{-1}}$ by medium dilution. Samples were afterwards exposed to darkness for 15 ${\textrm{min}}$ before measurement. The wavelengths used were 455 ${\textrm{nm}}$ for fluorescence excitation and 667–750 nm for fluorescence detection. The $F_v/F_m$ which represents the maximum quantum yield or maximum photosynthetic potential of PSII was calulated with equation (4):

4 $$\begin{aligned} F_v/F_m = (F_m - F_0)/F_m \end{aligned}$$

where $F_0$ is the minimum fluorescence yield determined after dark adaptation; $F_m$ is the maximal fluorescence measured after excitation by a 0.8 ${\textrm{s}}$ saturation light pulse with intensity of 3000 ${\upmu \textrm{mol} \, \textrm{m}^{-2} \, \textrm{s}^{-1}}$. $F_v$ is the difference between $F_m$ and $F_0$.

### 3D structure of biofilms

3D biofilm structure under different light conditions was monitored in *situ* and non-destructively by an inverted Zeiss LSM 700 Confocal Laser Scanning Microscope (CLSM, Carl Zeissmicroscopy GmbH, Jena, Germany). Microalgae biofilm were imaged using CLSM through Z-stack controlled by the Zen 10.0 software black edition (Carl Zeiss microscopy GmbH, Jena, Germany). All biofilm 3D structures were acquired through a LD Plan-Neofluar 20x0.4 Korr M27 objective with a 0.4 N.A. (numerical aperture). Each slice has a frame size of 512 $\times$ 512 pixels and image size of 638.9 $\times$ 638.9 ${\upmu \textrm{m}^2}$. Pixel size is 1.25 ${\upmu \textrm{m}}$. Each z-step is 3.94 ${\upmu \textrm{m}}$. One laser channel was applied to detect microalgal chlorophyll-a autofluorescence which was excited by 5-mW solid-state diode laser at 639 ${\textrm{nm}}$ and detected at 615 ${\textrm{nm}}$ after the long pass (CP) filter.

Biofilm of each flow-cell channel was measured in *situ* at five positions along the channel to obtain an average index of the biofilm structure. Measurements were carried out every 24 ${\textrm{h}}$ to follow the biofilm structural dynamics. Biofilm architecture was characterized by the following parameters: biovolume (${\upmu \textrm{m}^3\, \upmu \textrm{m}^{-2}}$), maximum thickness (${\upmu \textrm{m}}$), average thickness (${\upmu \textrm{m}}$), roughness coefficient (A.U.;ImageJ 1.48v software^52^, plug-in COMSTAT 2.1 from Technical University of Denmark^53^). It is worth noting that autofluorescence of cells is related to chlorophyll within chloroplast. However, to be in accordance with the terminology presented in most of the literature, we consider the increase of autofluorescence as cells proliferation, though autofluorescence does not quantify the cells.

### Biomass

#### Cell density

Biofilm cells were harvested from each channel by flushing Bristol medium through it, at least twice. Cell concentration was kept in the range of $1 \times 10^4$ to $6 \times 10^5$ ${\textrm{cells} \, \textrm{mL}^{-1}}$ by medium dilution and then measured by Guava easyCyte 5 flow cytometer (Millipore corporation 25801 Industrial Blvd Hayward, CA94545) with chlorophyll-a excitation at 488 ${\textrm{nm}}$ and fluorescence detection at 680 ${\textrm{nm}}$. Aerial cell density was obtained from total cell number in one channel divided by the surface of the substratum of the channel (0.24 ${\textrm{cm}^2}$).

#### Light transmittance

Light transmission through the biofilm was calculated daily based on the difference between PFD above and below the flow-cell (Eq. 5) measured by the light meter (LI-190/R; LI-COR Biosciences GmbH).

5 $$\begin{aligned} Light\;attenuation = \frac{I_{in} - I_{out}}{I_{in}} \times 100 \% \end{aligned}$$

where $I_{in}$ refers to incident light on the top of the flow-cell, $I_{out}$ refers to output light through the channel with biofilm (mean of three positions’ outputs along the channel).

### Growth rate

Biofilm specific growth rate was determined using light transmittance data.

The light transmittance in biofilms follows the Lambert–Beer Law:

6 $$\begin{aligned} I_{out} = I_{in} e^{-k\cdot X}, \end{aligned}$$

where X is the biomass (${\textrm{g}\, \textrm{m}^{-2}}$), *k* is the light extinction coefficient (${\textrm{m}^2 \, \textrm{g}^{-1}}$). Thus:

7 $$\begin{aligned} X = \frac{1}{k}ln \frac{I_{in}}{I_{out}}. \end{aligned}$$

Accordingly, the specific growth rate ($\mu _l$, ${\textrm{d}^{-1}}$) based on light transmittance is the maximum slope of the regression between $ln(ln\frac{I_{in}}{I_{out}})$ and time *t* (at least four data points were used). Number of replicates considered: 21, 8, 10, 15 under light intensity of 100, 200, 300, 500 ${\upmu \textrm{mol} \, \textrm{m}^{-2} \, \textrm{s}^{-1}}$, respectively.

### Statistics

Results are presented as mean and standard deviation. One-way and two-way ANOVA were proceeded by GraphPad prism 8.0 to test the statistical significance difference of means between different light regimes and time points. The level of significance was set at 0.05.

## Conclusions

In this study, we clearly demonstrated that *Chlorella vulgaris* biofilm 3D structure, physiology (cell size, chlorophyll content) are affected by light intensity. Our data confirm that sessile cells react to light intensity by adjusting the chlorophyll content (a decrease in chlorophyll per volume unit is observed with increased light) as in suspended cultures. In addition, for the first time, a regulation mechanism through cell organization and growth is highlighted in photosynthetic biofilms to cope with excess of light. Changes in physiology and photosynthetic activity were also reported when cells switched from suspended to sessile state, suggesting cell acclimation to the new lifestyle. Light conditions that maximize cell density of *Chlorella vulgaris* biofilms were identified (range between 100 and 300 ${\upmu \textrm{mol} \, \textrm{m}^{-2} \, \textrm{s}^{-1}}$). On the whole, this study gave some new insights into physiological and structural mechanisms occurring in photosynthetic biofilms which are required for biofilm-based system’s operation and optimization.

### Supplementary Information

Supplementary Information.

## Data Availability

The datasets generated and/or analyzed during the current study are not publicly available but are available from the corresponding author on a reasonable request.
