# Supplementary material for: The effect of light intensity on microalgae biofilm structures and physiology under continuous illumination
Source: Sci Rep. 2024 Jan 11;14:1151. doi: 10.1038/s41598-023-50432-6 (PMC10784318; doi:10.1038/s41598-023-50432-6)
Supplement: Supplementary file 1 — Supplementary Information. [file 41598_2023_50432_MOESM1_ESM.pdf]

The effect of light intensity on microalgae biofilm structures and physiology under continuous illumination

Yan Gao[1,2], Olivier Bernard[2], Andrea Fanesi[1], Patrick Perré[3], and Filipa Lopes[1,*]

[1]Université Paris-Saclay, CentraleSupélec, LGPM, Gif-sur-Yvette, 91190, France
[2]Université Nice Côte d'Azur, Inria Sophia Antipolis Méditerranée, Biocore, Valbonne, 06902, France
[3]Université Paris-Saclay, CentraleSupélec, LGPM, CEBB, Pomacle, 51110, France
[*]filipa.lopes@centralesupelec.fr

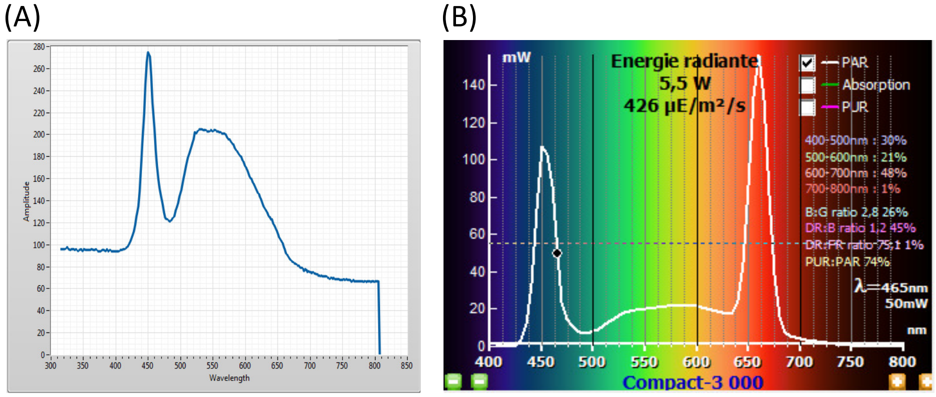

Figure S1. Light spectrum of the PSI MC1000 multicultivator (Photon systems instruments, Drásov, Czech Republic) (A), and of the Light Emitting Diode (Alpheus LED, Montgeron, France) (B).

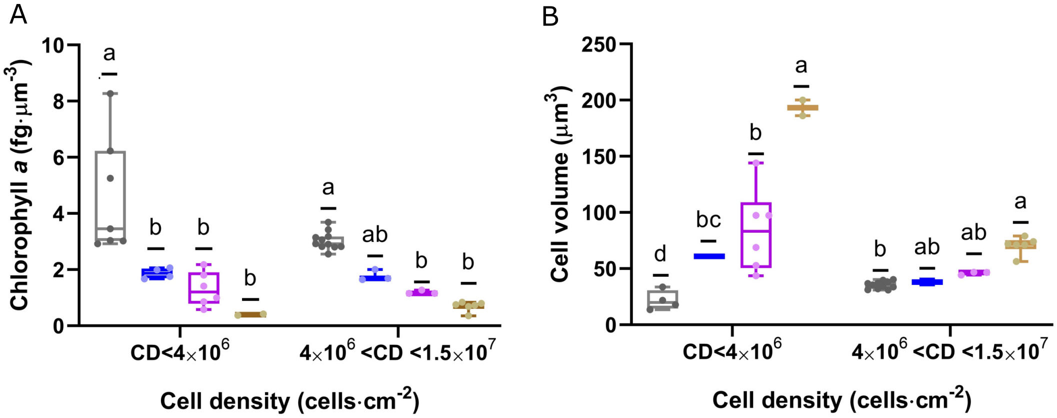

Figure S2. Light intensity effect on (A) chlorophyll-a content, (B) cell volume with different cell density domains. "CD" is the abbreviation of "cell density". Data are presented by box and whiskers with all data points from the minimum to the maximum. 100 $\mu mol \cdot m^{-2} \cdot s^{-1}$: grey; 200 $\mu mol \cdot m^{-2} \cdot s^{-1}$: blue; 300 $\mu mol \cdot m^{-2} \cdot s^{-1}$: magenta; 500 $\mu mol \cdot m^{-2} \cdot s^{-1}$: brown. Letters indicate the statistical significance among light intensities at each cell density domain analyzed by Two-way ANOVA at the level of $p<0.05$.

Photosynthesis and respiration measurements

Photosynthesis and respiration rates were carried out in the Chlorolab-2 liquid-phase system that use a Clark-type oxygen electrode (Hansatech, Norfolk, England) with 7-day biofilm cells harvested from flow-cell channel and re-suspended in fresh medium. Chlorophyll-a concentration was adjusted to the range of $5 \times 10^5 - 1 \times 10^6$ $\mathrm{pg \cdot mL^{-1}}$ by adjusting the cell concentration. Na_2HCO_3 was added to the system (final concentration: 3.33 $\mu\mathrm{mol \cdot mL^{-1}}$) in order to avoid carbon limitation. Before measurements, the cultures were dark adapted for 20 min. The evolution of oxygen production over time was measured during 20 min. The light intensity was set at the same value used for biofilm cultivation: 100, 200, 300, 500 $\mu\mathrm{mol \cdot m^{-2} \cdot s^{-1}}$ with biological replicate number of 1, 2, 1, 1, respectively. Oxygen concentration evolution over time in darkness was measured afterwards during 20 min. Net photosynthesis, P_{net}, (rate of O_2 production) during illumination and dark respiration, R, (rate of O_2 consumption) in darkness were calculated and normalized to chlorophyll-a content with equation (1),

Per milligram chlorophyll-a based O_2 evolution rate ($\mu\mathrm{mol_{O_2} \cdot mg_{chl}^{-1} \cdot h^{-1}}$)

$$O_{2,\, evolution\ rate}^{chl} = \frac{C_{O_2} - C_{O_1}}{C_{chl} \cdot C_c \cdot (t_2 - t_1)} \cdot 10^6 \cdot 3600 \quad (1)$$

where in C_{O_1} and C_{O_2} represent the O_2 concentration ($\mathrm{nmol \cdot mL^{-1}}$) at time t_1 (s) and t_2 (s); C_c is the cell concentration ($\mathrm{cell \cdot mL^{-1}}$) of the sample and C_{chl} is the chlorophyll-a content ($\mathrm{pg \cdot cell^{-1}}$).

The gross photosynthesis rate (P_{gross}) is then calculated as equation (2):

$$P_{gross} = P_{net} + R \quad (2)$$

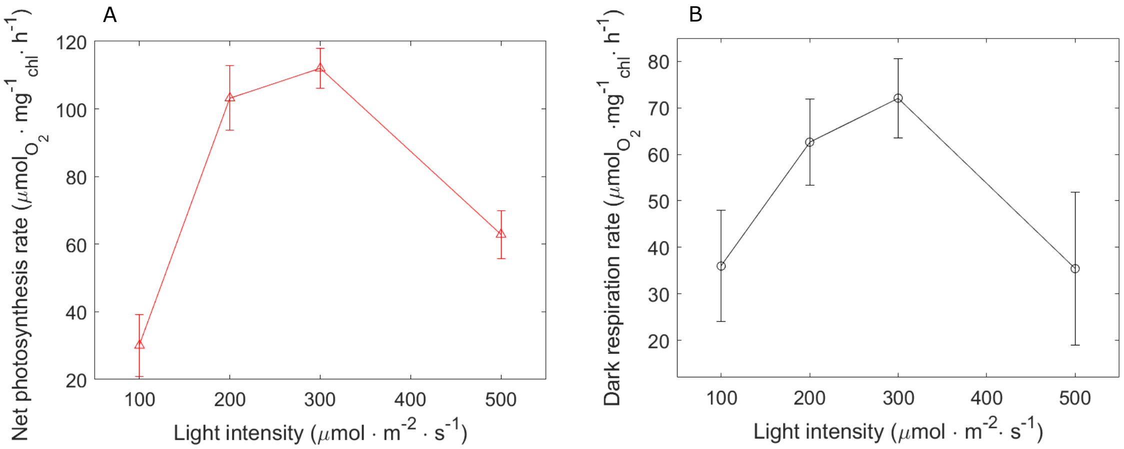

Figure S3. Photosynthetic and respiratory activities of biofilms under different light intensities at day 7. Data are presented as the mean with the standard deviation considering sampling points in 20 min illumination and 20 min darkness, respectively, extracted from Figure 4 presented in the main text.

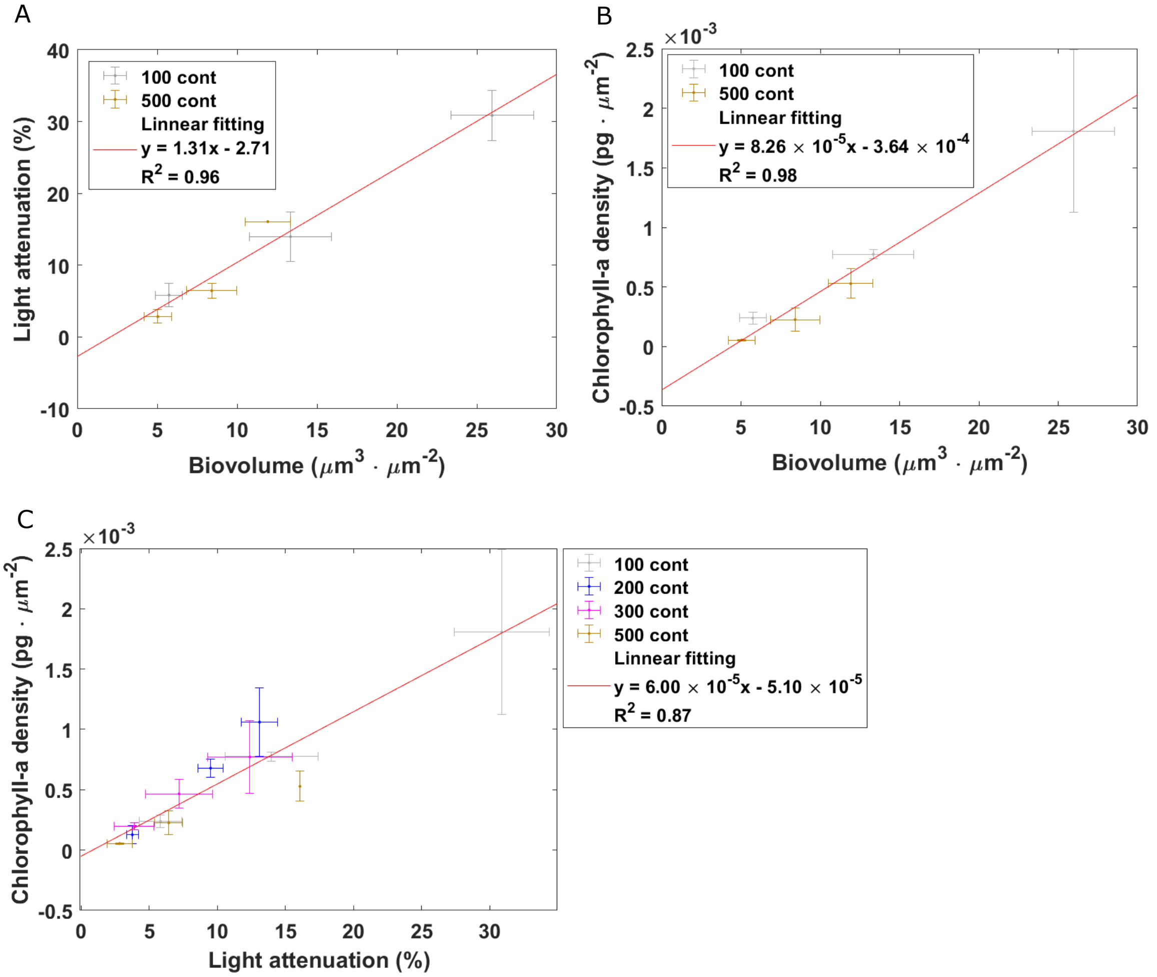

Figure S4. Biomass correlation among chlorophyll-a based parameters: (A) biovolume ($\mu m^3 \cdot \mu m^{-2}$), (B) light attenuation (%), and (C) chlorophyll-a density ($pg \cdot \mu m^{-2}$)
